# Phytohormone release by three isolated lichen mycobionts and the effects of indole-3-acetic acid on their compatible photobionts

**DOI:** 10.1007/s13199-020-00721-9

**Published:** 2020-10-22

**Authors:** Gregor Pichler, Wolfgang Stöggl, Daniela Trippel, Fabio Candotto Carniel, Lucia Muggia, Claudio Gennaro Ametrano, Tuğçe Çimen, Andreas Holzinger, Mauro Tretiach, Ilse Kranner

**Affiliations:** 1 Department of Botany, University of Innsbruck, Sternwartestraße 15, 6020 Innsbruck, Austria; 2 Department of Life Sciences, University of Trieste, Via Giorgieri 10, 34127 Trieste, Italy; 3 Grainger Bioinformatics Center, The Field Museum, 1400 S. Lake Shore Dr, Chicago, IL 60605 USA; 4 Department of Molecular Biology and Genetics, İzmir Institute of Technology, 35430 Izmir, Turkey

**Keywords:** Alga, Auxin, Fungus, Jasmonic acid, Lichen, Salicylic acid

## Abstract

Evidence is emerging that phytohormones represent key inter-kingdom signalling compounds supporting chemical communication between plants, fungi and bacteria. The roles of phytohormones for the lichen symbiosis are poorly understood, particularly in the process of lichenization, i.e. the key events which lead free-living microalgae and fungi to recognize each other, make physical contact and start developing a lichen thallus. Here, we studied cellular and extracellularly released phytohormones in three lichen mycobionts, *Cladonia grayi*, *Xanthoria parietina* and *Tephromela atra*, grown on solid medium, and the effects of indole-3-acetic acid (IAA) on their respective photobionts, *Asterochloris glomerata*, *Trebouxia decolorans*, *Trebouxia* sp. Using ultra-high-performance liquid chromatography coupled with tandem mass spectrometry (UHPLC-MS/MS) we found that mycobionts produced IAA, salicylic acid (SA) and jasmonic acid (JA). IAA represented the most abundant phytohormone produced and released by all mycobionts, whereas SA was released by *X. parietina* and *T. atra*, and JA was released by *C. grayi* only. With a half-life of 5.2 days, IAA degraded exponentially in solid BBM in dim light. When IAA was exogenously offered to the mycobionts’ compatible photobionts at “physiological” concentrations (as released by their respective mycobionts and accumulated in the medium over seven days), the photobionts’ water contents increased up to 4.4%. Treatment with IAA had no effects on the maximum quantum yield of photosystem II, dry mass, and the contents of photosynthetic pigments and α-tocopherol of the photobionts. The data presented may be useful for designing studies aimed at elucidating the roles of phytohormones in lichens.

**Electronic supplementary material:**

The online version of this article (10.1007/s13199-020-00721-9) contains supplementary material, which is available to authorized users.

## Introduction

Lichens are complex symbioses between a fungus, the “mycobiont”, with one or more species of green algae and/or cyanobacteria, the “photobiont”, also hosting a complex microbiota of bacteria and other microfungi (Honegger 1991; Grube and Berg 2009; Lutzoni and Miadlikowska 2009; Spribille et al. 2016; Cernava et al. 2017; Muggia and Grube 2018; Hawksworth and Grube 2020). The “signalling hypothesis” presented by Joneson and Lutzoni (2009) postulates that the transition to the symbiotic state involves chemical signals, which trigger metabolome re-arrangement prior to morphological changes. In support of this hypothesis, Joneson et al. (2011) showed that 11% and 12% of the algal genes and 16% and 28% of the fungal genes were overexpressed during the “pre-contact” and the “first contact stage”, respectively, concluding that the prospective symbionts start communicating prior to physical contact.

Phytohormones, including auxins, abscisic acid (ABA), jasmonates (JAs), salicylic acid (SA), brassinosteroids, cytokinins (CKs), ethylene, gibberellins (GAs) and strigolactones, are important signalling molecules in higher plants (Santner and Estelle 2009) with pivotal roles for plant development (Santner et al. 2009), and they may also play key roles in inter-kingdom signalling (Xu et al. 2015; Hughes and Sperandio 2008). Other organisms such as green algae, cyanobacteria (Lu and Xu 2015), bacteria and fungi are also capable of producing phytohormones and/or responding to these metabolites (Costacurta and Vanderleyden 1995; Tsavkelova et al. 2006; Chanclud and Mortel 2016). Hence, the term phytohormone is somewhat misleading, but widely used and accepted, and we will use it in the paper also with reference to fungal “phytohormones”.

Phytohormones are important players in fungal symbioses such as mycorrhiza. Phytohormones released by plant roots influence the metabolism and growth of mycorrhizal fungi and in turn, fungus-derived phytohormones affect root morphology, metabolism and growth of a plant (Gogala 1991; Pozo et al. 2015, for review). A broad range of mycorrhizal fungi produce the auxin, indole-3-acetic acid (IAA; Ek et al. 1983; Frankenberger and Poth 1987). In the mycorrhizal fungus *Tricholoma vaccinum*, the multidrug and toxic extrusion transporter Mte1 was shown to be responsible for IAA release, and the released IAA affected the morphology of the Hartig net (Krause et al. 2015). Moreover, mycorrhization (the process of mycorrhizal colonization of plant roots) was enhanced in an IAA-overproducing mutant of *Hebeloma* *cylindrosporum* compared to its wild type (Gay et al. 1994), providing further evidence for a role of IAA in mycorrhization. Furthermore, ABA was found in mycorrhizal fungi, for example in *Glomus* sp. (Esch et al. 1994), and SA and jasmonic acid (JA) were reported to be pivotal signalling compounds involved in the early stages of mycorrhiza establishment (Pozo and Azcón-Aguilar 2007). Jasmonates promote mycorrhization (Regvar et al. 1996) and fungal colonization was reported to be regulated by jasmonate signalling of the host plants (Herrera-Medina et al. 2008). In addition, CKs and GAs can enhance root colonization by mycorrhizal fungi (Cosme et al. 2016) and promote their entry into plant roots (Tominaga et al. 2020), but GAs are also known to suppress mycorrhiza formation, depending on fungus and plant species (Foo et al. 2013). Hause et al. (2007) reviewed that mycorrhization can be positively influenced by CKs, GAs and JA at low concentrations, and negatively by SA as well as by high concentrations of JA and ethylene. In summary, phytohormones represent important signalling compounds in mycorrhizae and therefore, it is reasonable to assume that phytohormones are also involved in the chemical cross-talk between other plant-fungus symbioses, such as the lichen symbiosis.

However, whereas much information is available on mycorrhizal symbioses, studies about the role of phytohormones in lichens are scarce. Epstein et al. (1986) and Ergün et al. (2002) detected phytohormones in several lichen species, including IAA, ABA, the cytokinin zeatin (ZT) and gibberellin A3 (GA3). Exogenous treatment of the photobiont *Trebouxia irregularis* with CKs (Bacškor and Hudák 1999) and of the mycobionts *Nephromopsis ornata*, *Myelochroa irrugans* and *Usnea longissima* with auxins increased growth (Wang et al. 2009; Wang et al. 2010). Interestingly, Meeßen et al. (2013) also found indole-3-carbaldehyde, an intermediate of IAA synthesis or degradation (Bandurski et al. 1995; Gazarian et al. 1998), in the culture medium of lichen-forming Trebouxiophyceae. Recently, we showed that photobionts isolated from the lichens *Cladonia grayi* Sandst., *Xanthoria parietina* (L.) Th. Fr. and *Tephromela atra* (Huds.) Hafellner release phytohormones into the environment; *Asterochloris glomerata* (Warén) Skaloud & Peksa released IAA, ABA and JA, *Trebouxia decolorans* Ahmadjian released IAA, ABA and *Trebouxia* sp. released IAA, ABA, JA, GA3 and indole-3-butyric acid (IBA; Pichler et al. 2020).

The two foremost aims of this study were firstly, to identify phytohormones produced by lichens mycobionts and secondly, to determine if phytohormones were released into their extracellular environment*. C. grayi*, *X. parietina* and *T. atra*, all members of the class Lecanoromycetes (Scherrer et al. 2005; Muggia et al. 2008; Armaleo and May 2009), were tested and eight phytohormones were analysed, ABA, GA_3_ and gibberellin A4 (GA_4_), IAA, IBA, JA, SA, and ZT. The third aim was to determine if phytohormones shared by these mycobionts are sufficiently stable to be perceived by other organisms after release into the extracellular space. Finally, we intended to obtain first insights into the potential effects of the fungal phytohormones on the physiology of the photobionts of the above-mentioned lichens, *A. glomerata*, *T*. *decolorans* and *Trebouxia* sp. To study the effects of mycobiont phytohormones on photobiont physiology, the following parameters were determined: photobiont growth, assessed by the increase in biomass, water content (WC), and maximum quantum yield of photosystem II (F_v_/Fm), this is a parameter linked to photosynthetic performance and frequently used to assess the health state of vascular plants, algae and lichens (Kalaji et al. 2014).

## Materials and methods

### Mycobionts

The axenically grown strain of *C. grayi* Sandst. (CBS 132746) was obtained from the Westerdijk Fungal Biodiversity Institute. Mycobionts of *X. parietina* (L.) Th. Fr. and *T. atra* (Huds.) Hafellner were isolated and kept as living cultures in the culture collections of the University of Trieste and the University of Graz, respectively. These mycobionts were chosen, because they represent well-studied model lichens, and based on one year of lab trials we found that cultures of their isolated mycobionts grew sufficiently fast for producing enough biomass for the intended experiments.

The identities of the mycobionts were confirmed by ITS sequencing (ITS1, 5.8S, ITS2) prior to experiments. PCR reactions were carried out as in Muggia et al. (2014) using the ITS1f and ITS4 primer pair (White et al. 1990). PCR products were visualized on 1.5% agarose gel and cleaned by Mag-Bind® TotalPure NGS magnetic beads (Omega Bio-Tek). Clean products and the reverse primer ITS4 were premixed and sent to Macrogen Europe for sequencing. Culture identity was confirmed at the species level by BLAST (sequence identity >99%). The ITS barcodes were submitted to NBCI GenBank with the following accession numbers: *Cladonia grayi* MT513230*, Xanthoria parietina* MT513231*, Tephromela atra* MT513232*.*

Fungal stock cultures were grown in 50 mL liquid Lilly-Barnett medium (LBM; pH 5.0) according to Yoshimura et al. (2002), modified by the addition of 20 g of sucrose L^−1^, referred to as “modified LBM” (mLBM). Cultures were grown in an incubator (Percival PGC-6HO, CLF Plant Climatics, Wertingen, Germany) at a 14 / 10 h “dim light” (20 μmol photons m^−2^ s^−1^) / dark cycle at 20 °C. To obtain the required amounts of fungal biomass for further experiments (which took 18 months), the liquid medium was renewed and the fungal biomass was homogenized every three months, as suggested by Yoshimura et al. (2002). For homogenizing, the total fungal biomass was transferred to 2 mL Eppendorf tubes and centrifuged (Sigma® 3–18 KS) at 800 x g at 15 °C for 2 min. The supernatant was removed with a syringe, the fungal biomass washed by adding 1 mL of distilled water (dH_2_O) and gently vortexed for 5 s. After a second centrifugation step at 800 x g at 15 °C for 2 min, the supernatant was removed and 1 mL dH_2_O was added to each tube together with a steel grinding ball (3 mm diameter) pre-cleaned with MeOH, and the fungal biomass was homogenized with a TissueLyser II (Qiagen, Düsseldorf, Germany) at 30 Hz. The homogenization time was optimized for each mycobiont species, using a final homogenization time of 20 s, 2 min and 4 min for *C. grayi*, *X. parietina* and *T. atra*, respectively. The obtained homogenous fungal suspension was used for further sub-culturing for biomass production, and for the phytohormone experiments described below.

### Photobionts

The algal strain (Cgr/DA1pho) of *A. glomerata* (Warén) Skaloud et Peksa was kindly provided by Daniele Armaleo (Duke University, USA), while the strains of *T. decolorans* Ahmadjian and *Trebouxia* sp. were isolated and kept as living cultures in the culture collection of the University of Trieste. Algal stock cultures were grown on solid *Trebouxia* medium (TM; 2% agar, pH 6.9) according to Ahmadjian (1973) in an incubator at the same conditions described above for mycobionts. To produce sufficient biomass for the intended experiments, sub-cultures were made every 14 days when the exponential growth phase was reached (see details in Pichler et al. 2020). Species identity was confirmed by ITS sequencing as described in Pichler et al. (2020) prior to experimentation. The ITS sequences were submitted to NBCI GenBank with the following accession numbers: *Asterochloris glomerata* MT603979, *Trebouxia decolorans* MT603980, and *Trebouxia* sp. MT603981.

### Determination of dry mass and preparation of suspensions of fungal inoculate

After homogenization, the fungal mass in the suspension was determined and expressed in mg fungal dry mass (DM) mL^−1^ (*n* = 3). For each mycobiont, 1 mL of fungal biomass was filtered through hydrophilic polytetrafluoroethylene (PTFE) membranes (25 mm diameter, pore size 0.45 μm, Omnipore™, Ireland) using a manual vacuum pump. For determination of DM, one set of filters was dried in an oven at 80 °C for approximately 3 h, until the DM was stable. The mean DM was calculated, and the fungal suspension adjusted to a final concentration of 4 mg fungal DM mL^−1^. 100 μL of each fungal suspension (n = 3 for each mycobiont) were examined using a microscope (Zeiss Axiovert 200 M, Jena, Germany) to assure that hyphal structures were largely intact, and photos were taken with a digital camera (Zeiss AxioCam HRc, Jena, Germany).

### Inoculation of fungal samples for phytohormone determination

To measure phytohormones in mycobiont cells and their release into the extracellular space, we chose to grow mycobiont cultures on solid growth media, which reflects the conditions in nature better than culturing lichen mycobionts in liquid media (Asplund and Wardle 2017). PTFE membranes (25 mm diameter, pore size 0.45 μm) were placed onto 10 mL of solid mLBM (2% agar, pH of 5) in Petri dishes (55 mm diameter, polystyrol (PS) Petri dishes, Rotilabo®, Germany) and inoculated with 500 μL of fungal suspension corresponding to approximately 4 mg fungal DM mL^−1^ each (exact weight recorded). The mycobionts were first grown on the filters in a growth chamber under dim light for 6 weeks, according to Yoshimura et al. (2002), then transferred onto 4 mL of nutrient-poor BBM (2% agar, pH 6.8; Bold 1949), in flat-bottom well plates (Corning Life Science, USA) and exposed to dim light for additional 7 days.

Before transfer to BBM, each sample was visually inspected for bacterial and fungal contamination with a stereomicroscope (Reichert, Austria) and photos of samples were taken with a digital full-frame camera (EOS 5D, Canon Inc., Japan) connected to a camera adapter (OM-Mount Photomicro Adapter L, Olympus®, Japan) and a photo eyepiece (NFK, 2.5 x LD 125, Olympus®, Japan). Photos were taken on the third day, and 6 and 7 weeks after inoculation. To determine concentrations of “cellular” phytohormones (i.e. intracellular and cell wall-bound phytohormones), fungal material was harvested after 7 weeks, washed with 1 mL liquid BBM, then centrifuged at 800 × g and 15 °C for 2 min, the supernatant removed and the fungal biomass freeze-dried (Zirbus VaCO 2, Zirbus technology GmbH, Germany) for at least 90 h according to Bailly and Kranner (2011); DM was determined with an analytical balance (XS 105, Mettler Toledo, Greifensee, Switzerland), and then the material was stored in a plastic box together with silica gel at −80 °C until phytohormone extraction. The agar medium was harvested in 5 mL Eppendorf tubes for determination of extracellular phytohormones (i.e. phytohormones that were released from fungal cells and accumulated in the medium within 7 d of growth on solid BBM), freeze-dried, weighed and stored as above.

### UHPLC-MS/MS analysis of cellular and released phytohormones

The phytohormones ABA, SA, JA, GA_3_, GA_4_, IAA, IBA and ZT were identified and quantified via ultra-high-performance liquid chromatography - mass spectrometry / mass spectrometry (UHPLC-MS/MS). Freeze-dried samples (*n* = 6 biological replicates) of cellular and extracellular phytohormones were extracted, processed and analysed as described in Pichler et al. (2020) for green microalgae, with minor modifications: 7 mg (instead of 5 mg) freeze-dried DM was used for cellular phytohormone quantification, and an isotopically labelled internal standard solution of deuterated indole-3-acetic acid (IAA-d5) at a concentration of 0.5 μM was used additionally for precise IAA quantification. Equipment for culturing, harvesting or UHPLC-MS/MS was rinsed three times with LC-MS-grade acetonitrile and ultra-pure water (UPW), and dried in a fume hood before use. Unless mentioned otherwise, all chemicals used were of highest purity (HPLC or LC-MS-grade) and obtained from Sigma-Aldrich or VWR Chemicals. Four replicates of processed blank samples, containing only the solid growth medium, were also analysed for each fungal species. In these blank samples, the phytohormones GA_3_, GA_4_, JA, IAA, IBA and ZT were not found. Trace amounts of ABA, slightly above detection limit, were measured in one of 12 processed blank samples, but no ABA was detected in any of the biological samples. Low amounts of SA were detected in all blank samples, and the mean SA values (of the four blanks) were subtracted from SA amounts found in the biological samples.

### Photometric analysis of IAA using Salkowski reagent

Because IAA was found to be released by all three mycobionts and accumulated in the solid BBM (see Results), we chose to study the response of their compatible photobionts to this particular phytohormone, and also investigated to which extent IAA degrades in solid BBM. The decrease in concentration of free IAA in solid BBM in dim light was measured with an adapted photometrical method described by Glickmann and Dessaux (1995) at time intervals over 21 days. A volume of 390 mL of solid BBM (2% agar) was prepared, and 10.56 mL of liquid BBM were mixed with 440 μL of EtOH containing 30 mg IAA mL^−1^. All solutions were adjusted to a pH of 6.80 with 1 M and 0.1 M NaOH. Only the solid BBM medium was autoclaved. The IAA solution was filtered through a sterile 0.2 μm hydrophilic glass fibre (GF) surfactant-free cellulose acetate (SFCA) filter (Minisart® NML Plus, Sartorius Stedim Biotech, Göttingen, Germany), and 10 mL were added to the autoclaved BBM, when its temperature decreased to 60 °C, resulting in a final concentration of 30 μg IAA mL^−1^. Control media (blanks) were processed exactly in the same way but without IAA. Each 55 mm Petri dish was filled with 10 mL of solid BBM used for photometrical assessment of IAA. To extract the solution containing either IAA or no IAA (blank) from the solid BBM, the medium was transferred to 2 mL Eppendorf tubes and centrifuged at 29,000 x g at 20 °C for 20 min. Supernatants of three technical replicates were pooled together to obtain sufficient volume for photometrical analyses, and the pH was measured with a pH electrode (SenTix® Mic, WTW™, Germany). The Salkowski reagent was prepared by mixing 2 ml of an aqueous 0.5 M FeCl_3_ solution with 41 mL UPW and 57 mL of 60% perchloric acid in a fume hood. The solution was kept in darkness at room temperature until use. 96 μL of each sample, 4 μL of EtOH and 200 μL of Salkowski reagent were mixed in 1.5 mL Eppendorf tubes (“sample mix”); 4 biological replicates (i.e. BBM medium from 55 mm Petri dishes containing IAA or no IAA), each with 4 technical replicates (i.e. multiple measurement of a biological replicate) were measured. Tubes were vortexed for 5 s and stored in darkness for 45 min prior to photometric measurement. The absorption maximum at 530 nm was confirmed for IAA dissolved in liquid BBM containing 4% of EtOH after adding 200 μL of Salkowski reagent. For photometric analyses, 200 μL of each sample mix were transferred to a 96 multi-well plate (Corning® 3635, Sigma-Aldrich, St Louis, MO, U.S.A.) and absorption was measured at 530 nm and 30 °C with a photometric plate reader (Synergy-HTX multi-mode reader, BioTek®Instruments, Winooski, VT, U.S.A.).

Data were obtained and analysed with the Gen5™ 2.07 software (BioTek® Instruments, Winooski, VT, U.S.A.). IAA sample contents were normalized to blanks (liquid BBM without IAA) and calculated by using a quadratic standard curve (R^2^ ≥ 0.99) constructed with freshly prepared standard solutions of IAA at concentrations of 0, 5, 10, 20, 30 and 40 μg mL^−1^ liquid BBM on the day of measurement. An IAA stock solution was prepared (1 mg mL^−1^ EtOH), diluted in liquid BBM and adjusted to an EtOH concentration of 4%. Mean values of biological replicates were calculated and plotted as a percentage (i.e. day 0 represents 100%) on day 0, 1, 2, 3, 4, 7, 14 and 21 to assess degradation and half-life (t_1/2_) of IAA in solid BBM medium under dim light.

### Exogenous IAA treatment of photobionts

After examining the degradation of IAA in solid BBM in dim light, this phytohormone was applied exogenously to each compatible photobiont at three concentrations, a) no IAA (control), b) physiological concentrations, i.e. concentration released by the respective mycobionts (0.001, 0.05 and 0.1 μM IAA for *C. grayi*, *X. parietina* and *T. atra*, respectively) and c) a “high” concentration of 1 μM IAA as used in recent papers (Piotrowska-Niczyporuk and Bajguz 2014; Kozlova et al. 2017).

For each species, two independent runs of these treatments were conducted using a randomized design, each run including five biological replicates, which resulted in a total of 10 biological replicates.

### Inoculation

For each algal species, 600 mg of fresh mass (FM) were taken after 2 weeks when they were in the exponential growth phase (Pichler et al. 2020), transferred to 5 mL Eppendorf tubes and washed by adding 2 mL of dH_2_O, prior to 5 s of vortexing and centrifugation at 500 x g at 15 °C for 2 min. Supernatants were removed with a syringe, 2 mL of dH_2_O was added and tubes were vortexed for approximately 10 s until a homogenous algal suspension was obtained. Algal DM within the suspension (mg DM mL^−1^) was determined in triplicates for each species by filtering 50 μL of each suspension through PTFE membranes (25 mm diameter, pore size 0.45 μm) with a manual vacuum pump. Filters were dried in an oven at 80 °C for approximately 3 h, until DM was stable (*n* = 3). Algal suspensions were adjusted to 40 mg algal DM mL^−1^ to obtain 2 mg algal DM per filter to be inoculated. Additionally, 3 × 10 μL of each algal suspension were examined with a microscope (Zeiss Axiovert 200 M, Jena, Germany) after adjustment to assure that algal cell structures were intact. Photos were taken with a digital camera (Zeiss AxioCam HRc, Jena, Germany).

### Preparation of petri dishes for exogenous IAA treatment

Prior to the inoculation of algae, different concentrations of fresh IAA solutions and water (for controls) were prepared daily and adjusted to a pH of 6.8. PTFE filters (25 mm diameter, pore size 0.45 μm) were placed onto 10 mL of solid BBM (2% agar, pH 6.8) in 55 mm Petri dishes. After sterile filtration of control and IAA solutions using 0.2 μm hydrophilic GF-SFCA filters, 50 μL of either 0 (control), 0.001, 0.05, 0.1 or 1 μM IAA were pipetted onto the solid BBM and left for 10 min to diffuse. Filters were inoculated with 50 μL of algal suspension containing 40 mg algal DM mL^−1^. Photobiont cultures were grown for seven days in a growth chamber under dim light and transferred to fresh growth medium with or without IAA every day. Filters supporting the cultures were transferred shortly before the start of each dark period to avoid that IAA degradation was accelerated due to light exposure, as described by Dunlap and Robacker (1988).

### *Chlorophyll a fluorescence* (*Chl*_a_*F*)

The maximum quantum yield of photosystem II (F_v_/Fm) of IAA-treated and -untreated photobionts was measured with a pulse-amplitude-modulated PAM-2500© fluorometer (Waltz, Effeltrich, Germany). Prior to F_v_/Fm measurements, algal samples were dark adapted for one hour and then placed 2 cm beneath a measuring optic fibre. The modulated light was turned on to obtain F_0_ (minimal Chl_*a*_F level). A saturating light pulse of 910 μmol photons m^−2^ s^−1^ for 300 ms was emitted to obtain F_m_ (transient maximum Chl_*a*_F level) and to calculate F_v_ (variable Chl_*a*_F level, i.e. F_m_ - F_0_) and F_v_/Fm (Genty et al. 1989). The F_v_/Fm was measured three times for each sample (*n* = 3 technical replicates for each of the 10 biological replicates) through the Petri dish lid to maintain sterility, including one-minute recovering breaks in-between, to avoid oversaturation. Samples were measured every second day.

### Sample harvesting, algal growth and water content

Prior to harvesting, samples were visually inspected for bacterial and fungal contamination with a stereomicroscope (Reichert, Austria). Algal biomass was harvested and transferred to 2 mL Eppendorf tubes (safe-lock Eppendorf Quality™, Eppendorf AG, Germany). Algal FM was determined with an analytical balance and samples were frozen in liquid nitrogen. After 90 h of freeze-drying (as described above) algal DM was measured and samples were stored on silica gel at −80 °C. Algal growth was expressed by the increase in biomass, expressed as FM and DM. Furthermore, FM and DM were used to calculate the percentage of algal WC as follows:

$$ WC\left(\% FM\right)=100\%\ast \frac{\left( FM- DM\right)}{FM} $$

### HPLC analysis of pigments and tocopherols

In addition, we studied if photobiont treatment with phytohormones affected the concentrations of plastid pigments, and of a major membrane-bound antioxidant, α-tocopherol. An adapted version of the method described by Remias et al. (2005) and modified by Buchner et al. (2017) was used for simultaneous HPLC analysis of photosynthetic pigments (absorbance at 440 nm) and tocopherols (fluorescence excitation: 295 nm, emission: 325 nm). For each extraction, 2.05 ± 0.62 mg algal DM were placed in 2 mL Eppendorf tubes. After adding two agate beads (5 mm diameter), samples were ground with a TissueLyser II at 25 Hz for 2 min. 200 μL of ice-cold N,N-dimethylformamide was added and samples were homogenized by tissue-lysing again at 25 Hz for 2 min, prior to centrifugation at 26,000 x g and 4 °C for 45 min. Supernatants were transferred to new 2 mL Eppendorf tubes, 100 μL of an aqueous MeOH solution (50 UPW: 50 MeOH, v:v) was added, and after vortexing for 5 s samples were centrifuged again at 26,000 x g, 4 °C for 45 min. 20 μL of supernatants were injected into an Agilent 1100 HPLC system equipped with a LiChrospher 100 RP-18 (125 × 4 mm, 5 μm) column (Agilent Technologies, Santa Clara, CA, U.S.A.). Pigments and tocopherols were identified and quantified using external standards. Authentic standards of chlorophyll a, b (Sigma-Aldrich, St. Louis, MO, U.S.A.), lutein, zeaxanthin (Carl Roth, Karlsruhe, Germany), antheraxanthin, neoxanthin, violaxanthin (DHI LAB Products, Hørsholm, Denmark), β-carotene (Merck, Darmstadt, Germany) and of α-, γ- and δ-tocopherol (Sigma-Aldrich, St. Louis, MO, U.S.A.) were used.

### Statistics

Numerical analyses were conducted with R (Version 3.5.1) and RStudio (Version 1.1.383.). Data were tested for normal distribution via QQ-plots and Shapiro-Wilk test. For cellular and released phytohormones of mycobionts, a non-parametric two-sided Mann-Whitney *U* Test with continuity correction (*p* value <0.01) was used to test for significant differences between cellular vs. released phytohormone concentrations. Significant differences in mycobiont and photobiont growth (FM, DM), photobiont water contents, photosynthetic pigment and tocopherol contents between controls and IAA treated samples were tested with the non-parametric Kruskal-Wallis-Test (*p* value ≤0.05) followed by Dunn’s post-hoc Test (*p* value ≤0.05) with Benjamini-Hochberg correction.

## Results

### Cultivation and assessment of cellular and released phytohormones of mycobionts

**Fig. 1:**
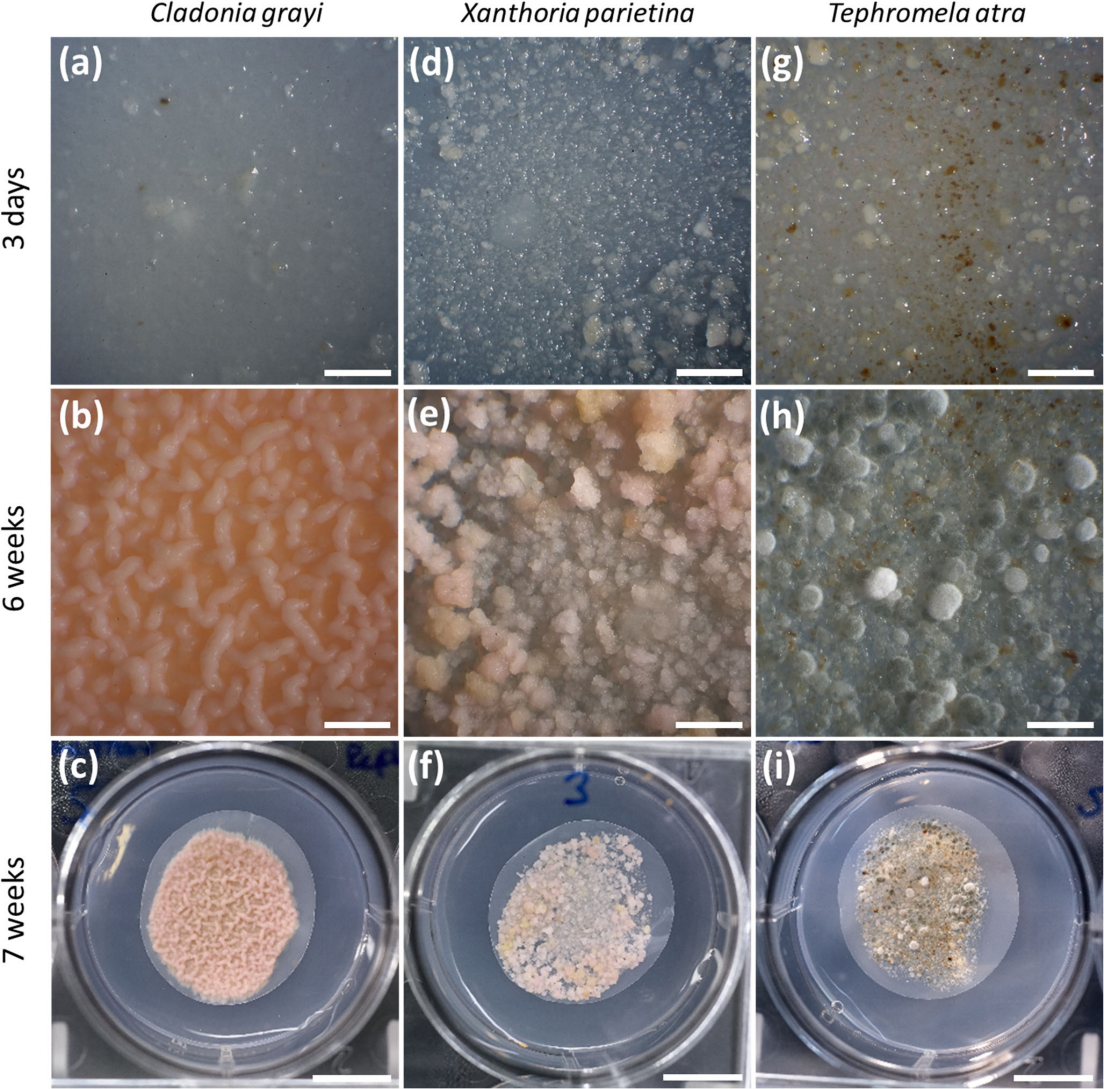
Phenotypes of isolated mycobionts grown in culture. Mycobionts were grown on PTFE filters and solid mLBM for six weeks, and then the filters with the mycobionts were transferred to solid BBM and left for an additional week, and then used for hormone analysis; **(a)** to **(c)** show *Cladonia grayi,* **(d)** to **(f)** *Xanthoria parietina* and **(g)** to **(i)** *Tephromela atra* cultures, three days, six weeks and seven weeks after inoculation; Scale bars: **(a, b, d, e, g, h)** 2 mm, **(c, f, i)** 1 cm

**Fig. 2:**
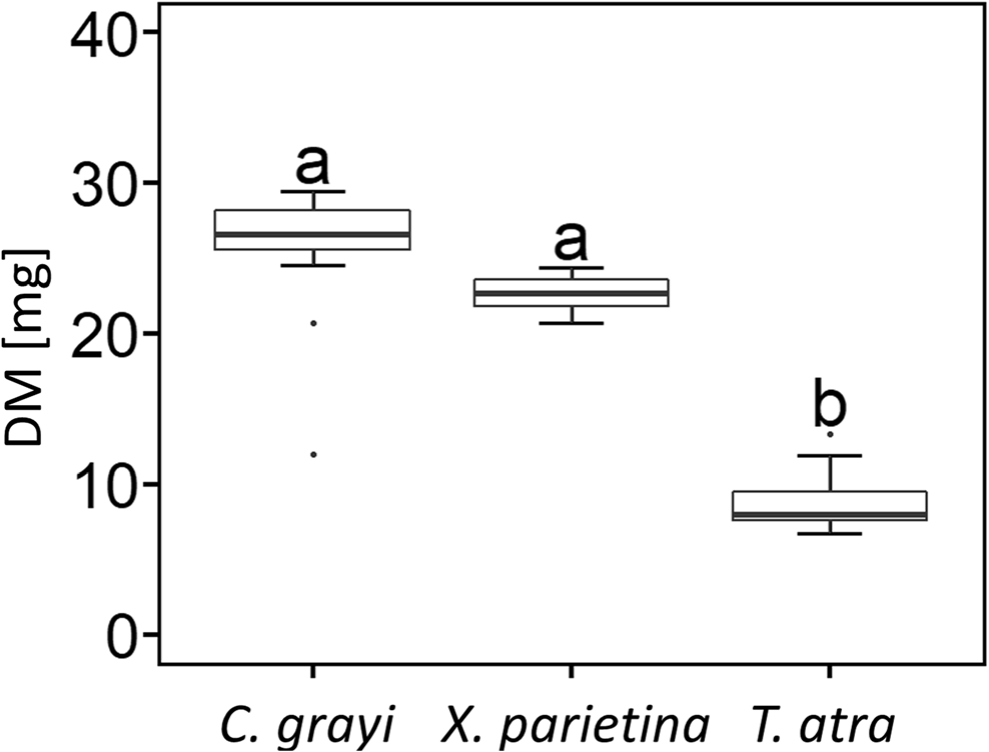
Increase in mycobiont dry mass (DM). Cultures of *Cladonia grayi*, *Xanthoria parietina* and *Tephromela atra* were grown for 7 weeks (to consider 6 weeks of pre-cultivation on solid mLBM plus 1 week after transfer to solid BBM). Box-plots show median, 25th and 75th percentiles, maxima, minima and outliers (dots); *n* = 13 to 14 biological replicates. Statistically significant differences, assessed with the Kruskal-Wallis-Test (*p* value <0.05) are marked by different letters above the box plots

All mycobionts developed abundant biomass six weeks after inoculation (Fig. 1a, d, g vs b, e, h), after which time they were transferred to solid BBM for an additional week prior to phytohormone assessment. *C. grayi* and *X. parietina* grew significantly faster (*p* value <0.05) than *T. atra*, assessed by the increase in DM, which was two- to threefold higher in the first two species after seven weeks of culturing (Fig. 2). In addition, mycobiont cultures showed different colours, i.e. white to pink, pink to yellow and grey to brown in *C. grayi*, *X. parietina*, and *T. atra*, respectively (Fig. 1).

**Fig. 3:**
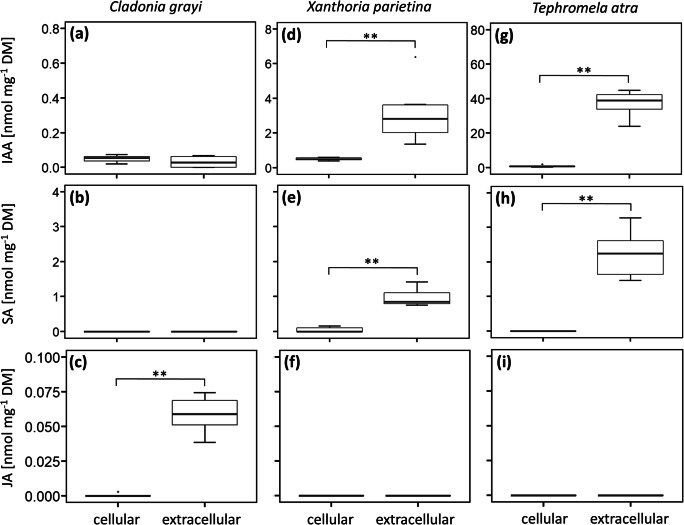
Cellular and extracellular amounts of the phytohormones indole-3-acetic acid (IAA), salicylic acid (SA) and jasmonic acid (JA) in **(a-c)** *Cladonia grayi*, **(d-f)** *Xanthoria parietina* and **(g-i)** *Tephromela atra* cultured for 7 weeks (details in Fig. 1); DM, dry mass. Box-plots show median, 25th and 75th percentiles, maxima, minima and outliers (dots), *n* = 6 biological replicates. Statistically significant differences according to a two-sided Mann-Whitney *U* test (*p* value <0.01) are marked with asterisks (**)

Of the phytohormones included in the UHPLC-MS/MS assay, IAA, SA and JA were detected in mycobionts and/or extracellular exudates that were allowed to accumulate in the agar medium over seven days before extraction. Only low amounts, both cellularly and extracellularly, of IAA and JA, but no SA, were found in *C. grayi*, and significant amounts of JA were found to be released (Fig. 3a-c), with concentrations being 122 times higher in exudates than in hyphae, normalized to fungal DM (*p* value <0.01; Fig. 3c). In addition, low amounts of IAA in *T. atra* (Fig. 3g), and of IAA and SA in *X. parietina* (Fig. 3d, e) were found cellularly, but considerable concentrations of these hormones were found to be released extracellularly by both species; normalized to fungal DM, 42 and six times, respectively, higher amounts of IAA were found in exudates of *T. atra* (Fig. 3g) and *X. parietina* (Fig. 3d) than cellularly, and around 21 times higher amounts of SA (*p* value <0.01) were released by *X. parietina* compared to cellular concentrations (Fig. 3e). In *T. atra*, SA was only detected in exudates (Fig. 3h). Jasmonic acid was not found in these two mycobionts, neither cellularly nor extracellularly (Fig. 3f, i). In summary, IAA was detected in all three mycobionts, representing the most abundant cellular and extracellularly released phytohormone. In addition, the concentrations of the detected phytohormones were considerably higher (six- to 42-fold higher) in the extracellular exudates compared to cellular concentrations, with the exception of IAA in *C. grayi*, which was found at the same concentrations cellularly and extracellularly.

**Fig. 4:**
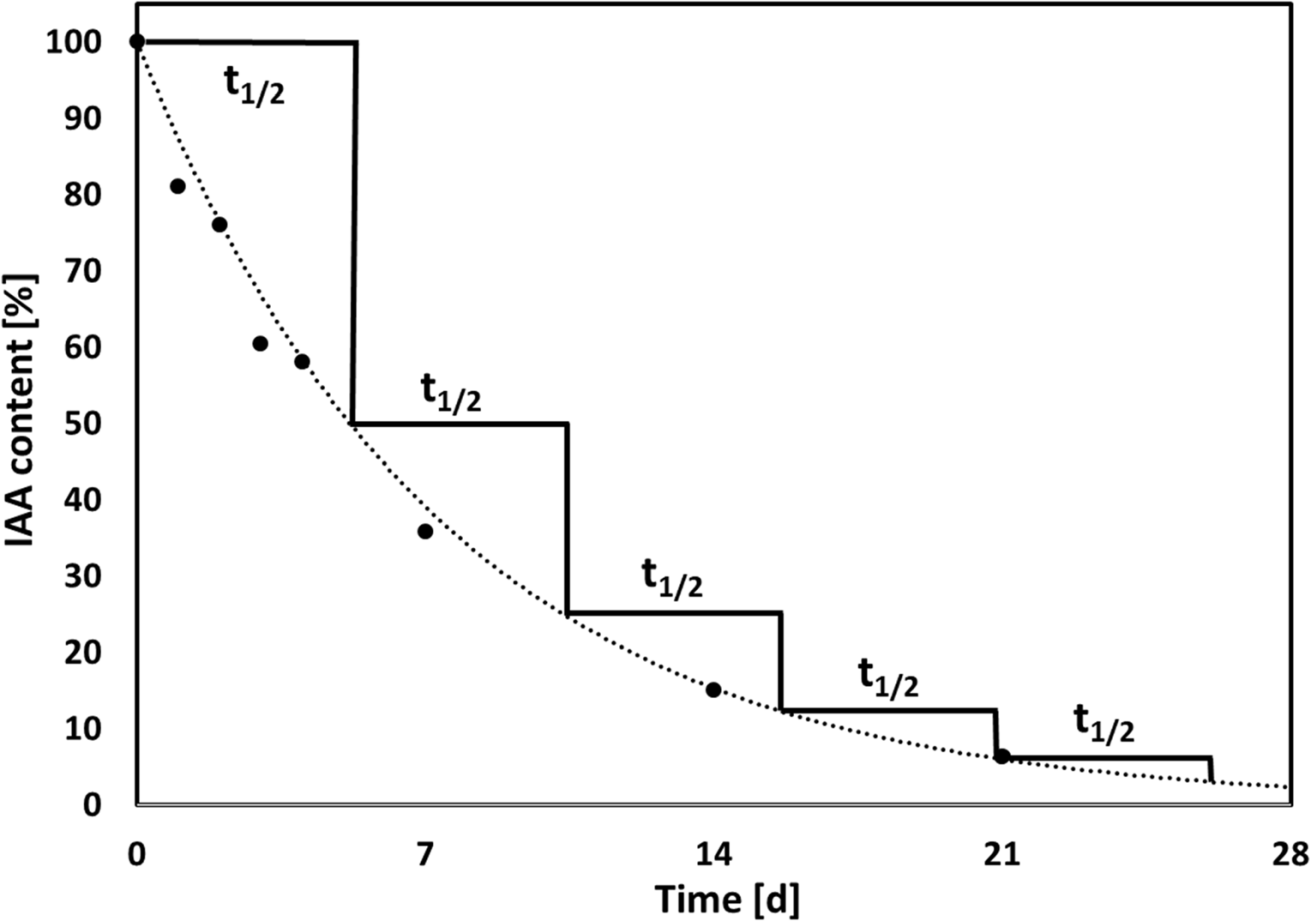
Degradation of indole-3-acetic acid (IAA) in solid BBM. The graph shows percentages of IAA retention in solid BBM (2% agar) in dim light over 28 days; black circles represent mean values, *n* = 4 biological replicates; the dotted line describes an exponential function (y = 100e^-0.134x^) with a coefficient of determination (R^2^) of 0.9959 and a half-life (t_1/2_) of 5.173 d

Because IAA was found in the exudates of all three mycobionts, we studied the effects of exogenous application of this phytohormone on their respective photobionts. To assure that sufficiently stable amounts of IAA were offered to the photobionts, we studied IAA degradation in solid BBM over time. The degradation of IAA in solid BBM (2% agar; pH 6.8) under dim light followed an exponential decrease, described with the function y = 100 e^-0.134x^ (coefficient of determination R^2^ = 0.996). The t_1/2_ of IAA, i.e. the time needed to observe a 50% decrease in the IAA content compared to the initial concentration (100%), was estimated to be 5.2 days (Fig. 4). Therefore, the filters supporting the photobiont cultures were transferred to fresh BBM supplemented with IAA every day.

*Effects of exogenous IAA treatment of photobionts on photosynthetic performance, growth, water contents, pigments and tocopherol contents.* After determining the degradation pattern of IAA in BBM, IAA was exogenously applied (see Methods) to the compatible photobionts of the three IAA-releasing mycobionts to test the effects of IAA on photobiont growth (assessed by FM and DM), WC, F_v_/Fm, pigment and tocopherol contents.

The F_v_/Fm values of IAA-treated *A. glomerata*, *T. decolorans* and *Trebouxia* sp. ranged from 0.55 to 0.71, 0.52 to 0.64 and 0.51 to 0.64, respectively, which did not differ from values in untreated controls, and no significant differences were found among IAA treatments (i.e. physiological and high IAA concentration), revealing that exogenous treatment with IAA did not affect photosynthetic performance, and hence, photobiont health (Fig. S1).

**Fig. 5:**
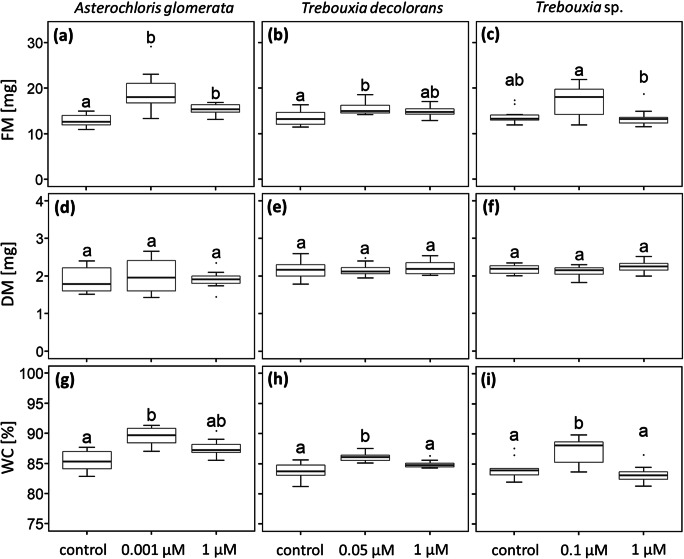
Growth and water contents of isolated photobiont cultures exposed to different IAA concentrations. Panels **(a)** to **(c)** show fresh mass (FM), **(d)** to **(f)** dry mass (DM), and **(g)** to **(i)** the water contents (WC) of untreated (controls) *Asterochloris glomerata*, *Trebouxia decolorans* and *Trebouxia* sp. photobiont cultures, respectively, and of cultures exposed to IAA exogenously applied either at physiological concentrations (0.001, 0.05 and 0.1 μM, defined by the IAA concentrations released extracellularly by their respective mycobionts) or “high” IAA concentrations (1 μM). Box-plots show median, 25th and 75th percentile, maxima, minima and outliers (dots); *n* = 10 biological replicates. Statistically significant differences, assessed with the Kruskal-Wallis-Test (*p* value <0.05) are marked by different letters above the box plots

Compared to controls, the FM of *A. glomerata* (Fig. 5a) and *T. decolorans* (Fig. 5b) increased by 42.6% and 13.7%, respectively (significant at *p* value <0.05) when cultures were treated with IAA at physiological concentrations. In *A. glomerata* FM also increased significantly (*p* value <0.05) by 21.4% after treatment with high IAA of 1 μM. However, exogenous IAA treatment did not affect the DM of any photobiont (Fig. 5d-f), indicating that the increase in FM was due to elevated WCs. Compared to controls, the WCs of *A. glomerata*, *T. decolorans* and *Trebouxia* sp. increased significantly (*p* value <0.05) by 4.4%, 2.3% and 4.1%, respectively, after treatment with physiological IAA concentrations (Fig. 5g-i).

No effects of any IAA concentration on the contents of photosynthetic pigments and α-tocopherol were observed in any of the photobionts, when data were normalized to DM, with the exception of violaxanthin in *Trebouxia* sp. treated with IAA at physiological concentration of 0.1 μM, which decreased slightly (*p* value <0.05) compared to controls (Table S1; Fig. S2c).

## Discussion

Within seven weeks, the mycobionts isolated from the two foliose lichens, *C. grayi and X. parietina*, produced between two to three times more biomass compared to the mycobiont of the crustose lichen *T. atra* (Fig. 2), in agreement with the generally faster growth reported for foliose lichens compared to crustose ones (Armstrong and Bradwell 2010, 2011). Reports about techniques to culture isolated lichen mycobionts are scarce (Yoshimura et al. 2002), and producing sufficient mycobiont biomass for physiological experiments is time consuming and often unsuccessful (Muggia et al. 2017). We showed that solid LBM supplemented with 2% sucrose is very apt for mycobiont growth (Fig. 2). The three mycobiont cultures had different colours (Fig. 1), according to the secondary metabolites, such as depsides, depsidones, anthraquinones and/or melanins, which are produced by lichens (Solhaug et al. 2003; Calcott et al. 2018), but can also be observed in cultured mycobionts (Fahselt 1994). Grayanic acid, 4-O-demethylsphaerophorin and 4-O-demethylgrayanic acids were identified in mycobiont cultures of *C. grayi* (Culberson and Armaleo 1992), lichen thalli of *X. parietina* typically contain parietin (Solhaug and Gausla 1996), and *T. atra* thalli contain atranorin, α-alectoronic acid and α-collatolic acid (Millot et al. 2008), reflected by the white to pink, pink to yellow and grey to brown colour of the *C. grayi*, *X. parietina* and *T. atra* mycobiont cultures (Fig. 1).

The limited studies on lichen phytohormones available revealed that lichens are capable of producing IAA, ZT, ethylene, GA_3_ (Epstein et al. 1986; Dietz and Hartung 1998; Ott et al. 2000; Ergün et al. 2002) and ABA; the latter received attention due to its involvement in water stress response and was also found in podetia of the lichen *Baeomyces rufus* (Dietz and Hartung 1998, 1999). Exogenous ABA treatment also improved the capability of the lichen *Peltigera polydactylon* to recover after long-term desiccation (Beckett et al. 2005). Furthermore, Schieleit and Ott (1996) showed that *C. rangiferina* and *Cetraria islandica* and their isolated myco- and photobionts produced ethylene. However, with the exception of the pioneering study by Schieleit and Ott (1996) it remains unclear which symbiont is responsible for the production of individual phytohormones. We recently showed that IAA, ABA, JA, GA3, IBA and ZT are produced and released by the compatible photobionts of *C. grayi, X. parietina* and *T. atra* (Pichler et al. 2020), and in the present study, we report on cellular and extracellular phytohormone release by their corresponding mycobionts. We found low cellular concentrations of IAA and JA in *C. grayi*, IAA and SA in *X. parietina* and IAA in *T. atra*, compared to the substantially higher concentrations found in their exudates; in addition, *T. atra* also released SA (Fig. 3). Further evidence for mycobiont-derived phytohormones was presented by Dietz and Hartung (1998), who detected ABA in podetia of the lichen *Baeomyces rufus*. Podetia are three-dimensional thalline stem-like structures derived by the proliferation of an apothecium, bearing hymenial discs and sometimes conidiomata, and in *B. rufus,* podetia are devoid of photobionts. Therefore, it is likely that the *B. rufus* mycobiont produces ABA, although co-symbiotic fungi (Mark et al. 2020) or bacteria (Cernava et al. 2017) present in lichen thalli could also be involved in ABA production, which was shown to occur in free-living bacteria (Karadeniz et al. 2006; Cohen et al. 2008) and fungi (Hirai et al. 2000; Hartung 2010). We did not find ABA in the mycobionts used in our study, whereas SA, which has not been reported so far for lichens or isolated photo- and mycobionts, was detected in *X. parietina* hyphae. Another phytohormone, JA, was found in *C. grayi* hyphae. Exogenously applied JA stimulated the growth of the isolated *Nephromopsis ornata* mycobiont (Wang et al. 2010), although the concentration of 1 μM JA used by these authors might have been outside the physiological range, at least judging by our findings that JA accumulated in the growth medium within a week was found only in the picomolar range. Exogenous application of IAA, the phytohormone found in all three mycobionts studied (Fig. 3a, d, g), also enhanced growth of the *Myelochroa irrugans* mycobiont (Wang et al. 2010). IAA was found in all mycobionts (Fig. 3a, d, g), is also produced by mycorrhizal fungi (Ek et al. 1983; Barroso et al. 1986) and lichen photobionts (Pichler et al. 2020), and hence, could play a role in chemical communication if released into the extracellular space and perceived by other organisms. It was outside the remits of this study to investigate the complex pathways of inter-kingdom signalling, but we aimed to provide baseline data regarding the release of phytohormones, which are known to play a role in mycorrhization (Pozo et al. 2015), by lichen mycobionts.

We found that IAA was released by all three mycobionts studied; in addition, *C. grayi*, which associates with *A. glomerata*, also released JA, and *X. parietina* and *T. atra*, which associate with *Trebouxia* spp., released SA (Fig. 3). Expressed on mycobiont DM, the concentrations of IAA, SA and JA accumulating over 7 days in the medium were up to 122 times higher than cellular concentrations, except for IAA released by *C. grayi,* which was found in the medium at the same concentrations as in hyphae. Indole signalling appears to regulate biofilm formation by *Escherichia coli* (Di Martino et al. 2003) and exogenously applied IAA can influence bacterial gene activity and can be catabolised by bacteria as a carbon source (Donoso et al. 2016). Lichen-associated bacteria are also able to release various volatile organic compounds, which inhibited sporulation and mycelium proliferation of the fungus *Botrytis cinerea* and reduced growth and metabolic activity of the bacterium *E. coli*, suggesting a potential role in pathogen defence (Cernava et al. 2015).

As IAA, a phytohormone with known roles in plant-microbe interactions (for review see Spaepen and Vanderleyden 2010), was found to be released by all three mycobionts, we studied if IAA was sufficiently stable after release to be perceived by the photobionts. Dunlap and Robacker (1988) showed that salts and light accelerate IAA degradation in Murashige-Skoog’s medium, and Nissen and Sutter (1990) reported that autoclaving facilitates IAA degradation. We found that IAA decreased exponentially with a half-life of 5.2 days (Fig. 4), in agreement with data reported for Murashige-Skoog’s (MS) medium (Nissen and Sutter 1990), a growth medium commonly used in plant tissue culture (Murashige and Skoog 1964). On the other hand, the three lichen photobionts *A. glomerata*, *T. decolorans* and *Trebouxia* sp., are able to release IAA extracellularly themselves (Pichler et al. 2020). Similarly, mycorrhizal fungi and plant roots are both capable of producing the same signalling compounds (Krause et al. 2015; Pozo et al. 2015). Therefore, to minimize variations in IAA concentration by IAA degradation over time and IAA production by the photobionts, cultures were transferred to fresh growth medium every day. Although the classical auxin signalling pathways discovered in higher plants appear to be absent in green microalgae (Lau et al. 2009), several studies showed that IAA can affect the metabolism of green microalgae (Gupta and Agrawal 2004; Bajguz and Piotrowska-Niczyporuk 2013) and therefore, alternative auxin signalling pathways may exist (Lau et al. 2009). Fungi use IAA for pathogenic or symbiotic interaction with other fungi, bacteria, plants and/or algae (Fu et al. 2015). Here, we offered exogenous IAA to lichen photobionts at physiological concentrations, i.e. as released by their compatible mycobionts (Fig. 3a, d, g), and at higher concentrations of 1 μM as used by previous authors (Piotrowska-Niczyporuk and Bajguz 2014; Kozlova et al. 2017). Several studies showed that IAA treatment enhanced growth and pigment contents of green algae. For example, IAA treatment increased the contents of chlorophylls and carotenoids in *Scenedesmus quadricauda* (Kozlova et al. 2018), of chlorophyll, proteins and monosaccharides in *Chlorella vulgaris* (Bajguz and Piotrowska-Niczyporuk 2013), and enhanced growth in *Chlorella* sp., *Dunaliella salina, Porphyridium cruentum*, and *Scenedesmus obliquus*, (Li et al. 2007; Salama et al. 2014). By contrast, in the present study, pigment and α-tocopherol contents were not affected by IAA treatment, with the exception of violaxanthin in *Trebouxia* sp., which slightly decreased when treated with 0.1 μM IAA (Fig. S2c). Importantly, no changes in photobiont growth and F_v_/Fm were observed (Fig. S1), indicating that the treatment did not exert harmful effects on the photobionts.

However, when treated with physiological IAA concentrations, the WCs of all three photobiont cultures increased significantly by up to 4.4% (Fig. 5g-i). It is known for a long time that IAA enhances the water uptake in cells of higher plants (Ordin et al. 1956) by changing the elastic properties of the cell wall (Zimmermann et al. 1976), but there are no such reports for micro-algae, with the exception of Kozlova et al. (2017), who showed that IAA treatment of *S. quadricauda* increased cell size. In summary, we showed that lichen mycobionts can produce and release phytohormones into the extracellular environment. IAA was found to be released by all three mycobiont species, with sufficient stability to be available and perceivable by other organisms, and significantly increased the WCs of the mycobionts’ compatible photobionts.

### Concluding remarks and outlook

Fungal symbioses such as lichens and mycorrhizae represent complex biological systems. The fact that both of the main symbionts are capable of producing the same signalling compounds complicates studies into the effects a compound released by one of the symbionts has on the other (disregarding their microbiota, which adds yet another level of complexity). Auxins are pivotal signalling molecules involved in the regulation of plant water status (Leyser 2011). However, their role in regulating lichen water relations has not been investigated and our study may help designing further experiments. Moreover, prior to using molecular tools for studying the role of phytohormones in initiating a symbiosis, such as producing mutants with a modified ability to synthesize a phytohormone or mutants with altered hormonal signalling, it must be known which phytohormones to target. Therefore, the work presented is envisaged to provide valuable baseline information on the occurrence of IAA, SA and JA in three lichen mycobionts, and the concentrations of these phytohormones after release into the environment, supporting future research aimed at clarifying the roles of phytohormones in inter-kingdom signalling.

## Electronic supplementary material

Fig. S1 Chlorophyll fluorescence of photobionts. Panels show means ± SD (*n* = 10 biological replicates) of F_v_/Fm for **(a)** *Asterochloris glomerata*, **(b)** *Trebouxia decolorans* and **(c)** *Trebouxia* sp. Dashed lines show untreated cultures (controls), solid lines show cultures exposed to IAA exogenously applied at physiological concentrations (0.001, 0.05 and 0.1 μM, defined by the IAA concentrations released extracellularly by their respective mycobionts); dotted lines show “high” IAA concentrations (1 μM). (PNG 9695 kb)

High resolution image (TIF 991 kb)

Fig. S2 Contents of violaxanthin levels of isolated photobiont cultures exposed to exogenous IAA. Panels **(a)** to **(c)** show violaxanthin levels of untreated (controls) *Asterochloris glomerata*, *Trebouxia decolorans* and *Trebouxia* sp., respectively, and of cultures exposed to IAA exogenously applied either at physiological concentrations (0.001, 0.05 and 0.1 μM, defined by the IAA concentrations released extracellularly by their respective mycobionts) or elevated IAA concentrations (1 μM). Box-plots show median, 25th and 75th percentiles, maxima, minima and outliers (dots); n = 10 biological replicates. Statistically significant differences, assessed with the Kruskal-Wallis-Test (*p* value <0.05) are marked by different letters above the box plots. (PNG 9695 kb)

High resolution image (TIF 1019 kb)

Table S1 Contents of photosynthetic pigments of isolated photobiont cultures exposed to exogenously offered IAA. Data show mean ± SD values of chlorophyll a (Chl a) and b (Chl b), lutein (L), zeaxanthin (Z), antheraxanthin (A), violaxanthin (V), α-carotin (α-C) and β-carotin (β-C), and α-tocopherol (α-T) levels of *Asterochloris glomerata*, *Trebouxia decolorans* and *Trebouxia* sp. of untreated cultures (controls), and of cultures exposed to IAA exogenously applied either at physiological concentrations (0.001, 0.05 and 0.1 μM, defined by the IAA concentrations released extracellularly by their respective mycobionts) or “high” IAA concentrations (1 μM); n = 10 biological replicates. Statistically significant differences, assessed with the Kruskal-Wallis-Test (*p* value <0.05) are indicated by different superscript letters. (DOCX 25 kb)
