# Supplementary figures and images for: Phytohormone release by three isolated lichen mycobionts and the effects of indole-3-acetic acid on their compatible photobionts
Source: Symbiosis. 2020 Oct 22;82(1):95–108. doi: 10.1007/s13199-020-00721-9 (PMC7671983; doi:10.1007/s13199-020-00721-9)

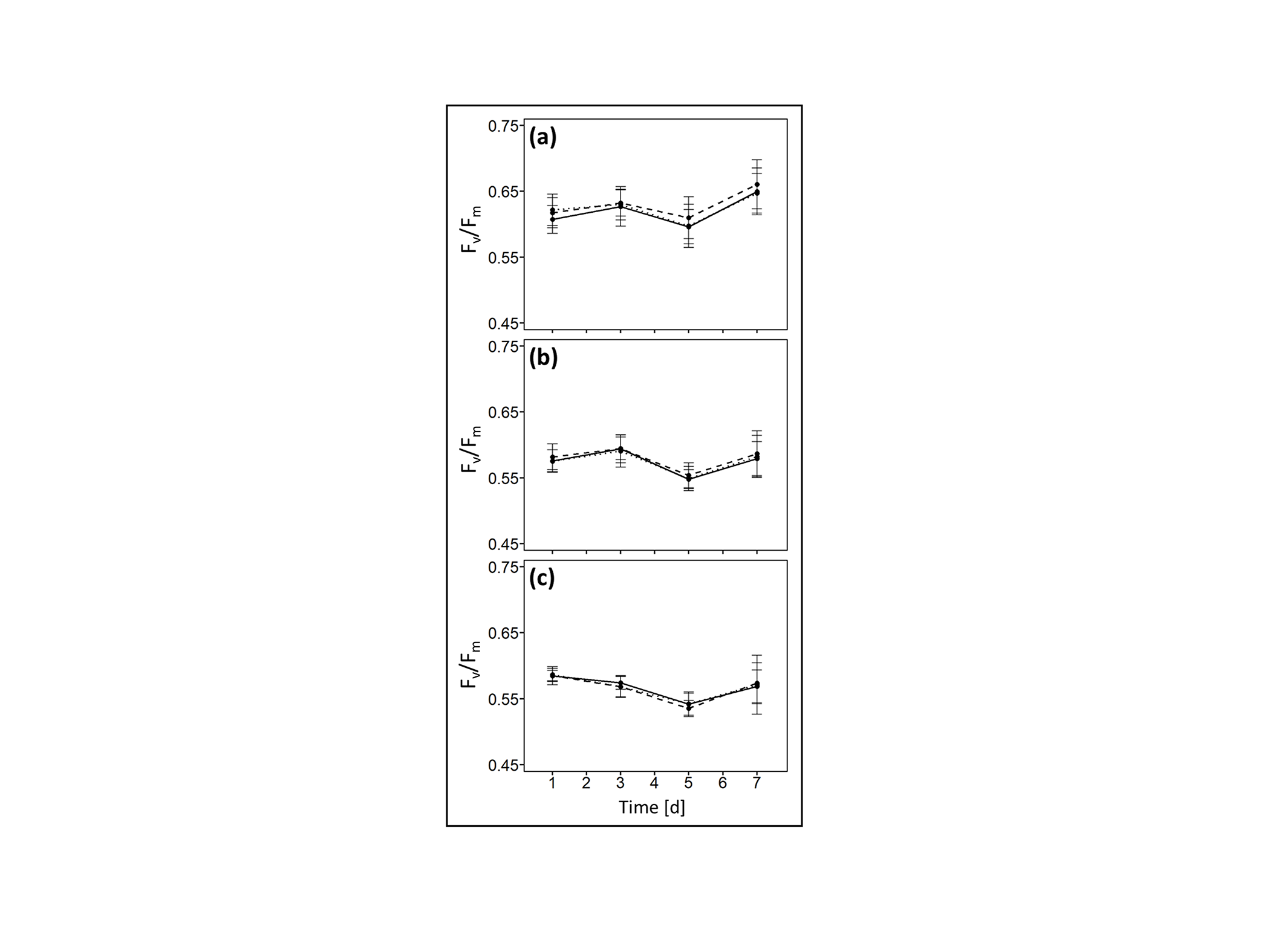

Supplement: Supplementary file 1 — Chlorophyll fluorescence of photobionts. Panels show means ± SD (n = 10 biological replicates) of Fv/Fm for (a) Asterochloris glomerata, (b) Trebouxia decolorans and (c) Trebouxia sp. Dashed lines show untreated cultures (controls), solid lines show cultures exposed to IAA exogenously applied at physiological concentrations (0.001, 0.05 and 0.1 μM, defined by the IAA concentrations released extracellularly by their respective mycobionts); dotted lines show “high” IAA concentrations (1 μM). (PNG 9695 kb) [file 13199_2020_721_Fig6_ESM.png]

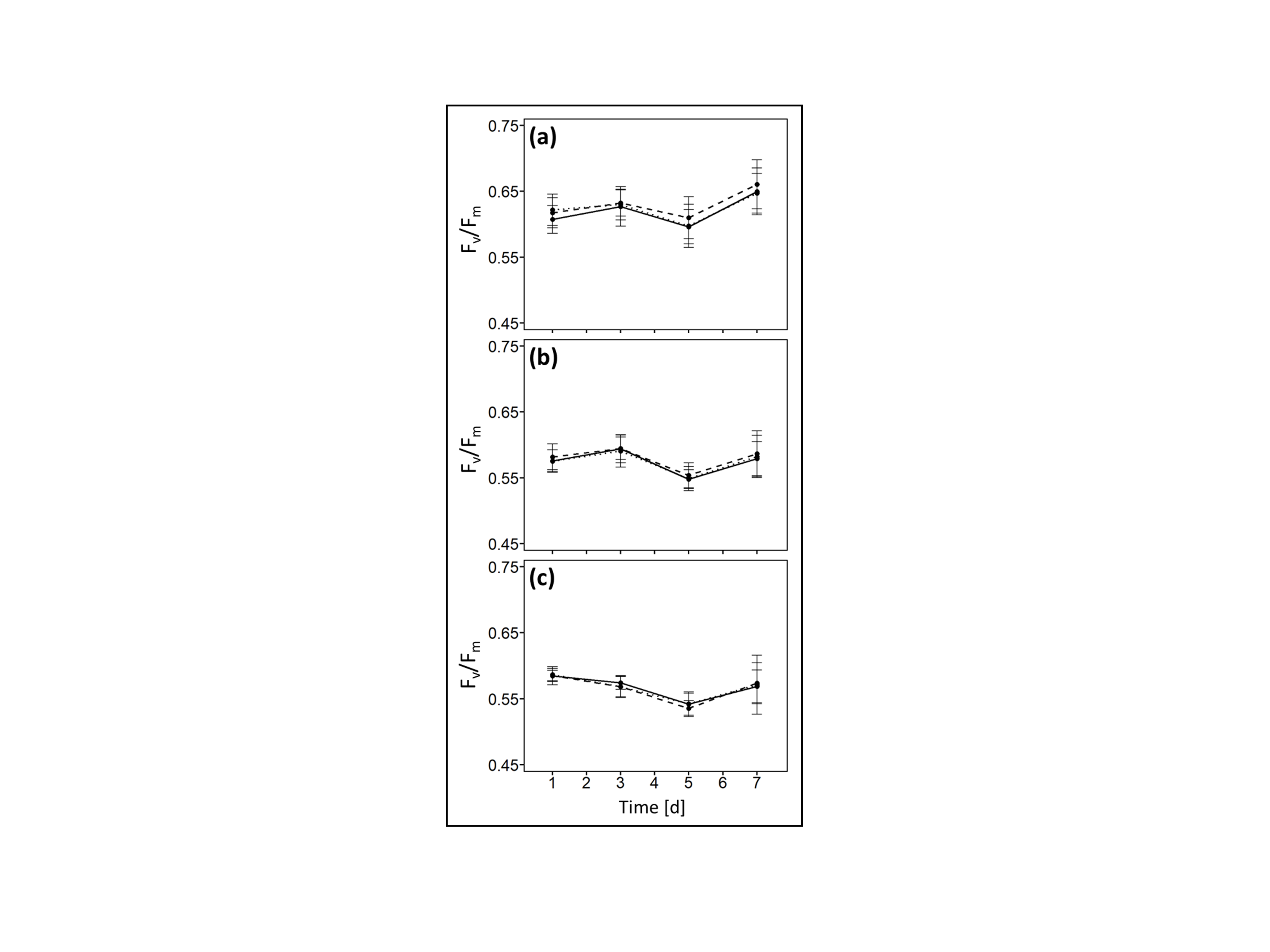

Supplement: Supplementary file 2 — High resolution image (TIF 991 kb) [file 13199_2020_721_MOESM1_ESM.tif]

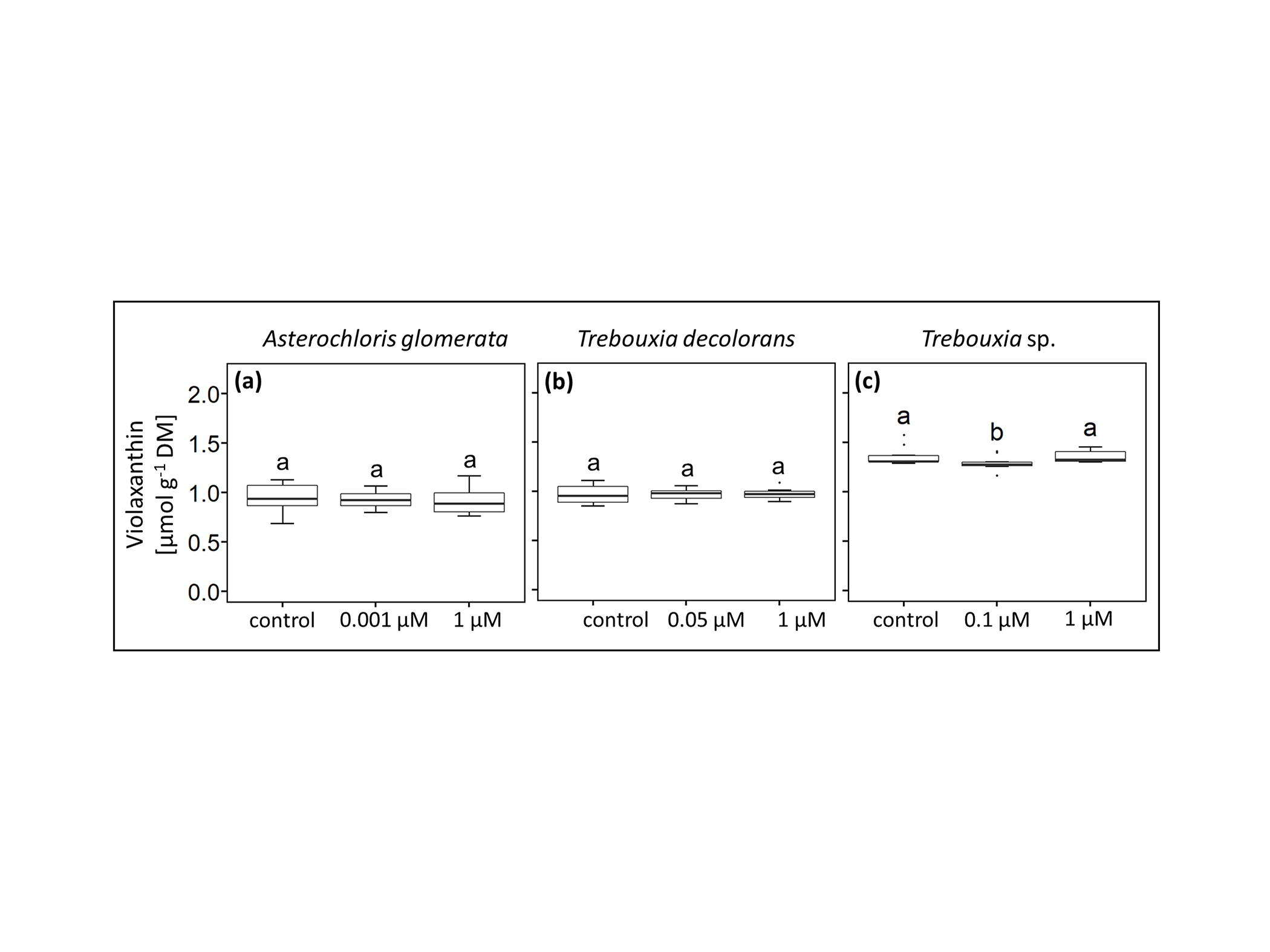

Supplement: Supplementary file 3 — Contents of violaxanthin levels of isolated photobiont cultures exposed to exogenous IAA. Panels (a) to (c) show violaxanthin levels of untreated (controls) Asterochloris glomerata, Trebouxia decolorans and Trebouxia sp., respectively, and of cultures exposed to IAA exogenously applied either at physiological concentrations (0.001, 0.05 and 0.1 μM, defined by the IAA concentrations released extracellularly by their respective mycobionts) or elevated IAA concentrations (1 μM). Box-plots show median, 25th and 75th percentiles, maxima, minima and outliers (dots); n = 10 biological replicates. Statistically significant differences, assessed with the Kruskal-Wallis-Test (p value <0.05) are marked by different letters above the box plots. (PNG 9695 kb) [file 13199_2020_721_Fig7_ESM.png]

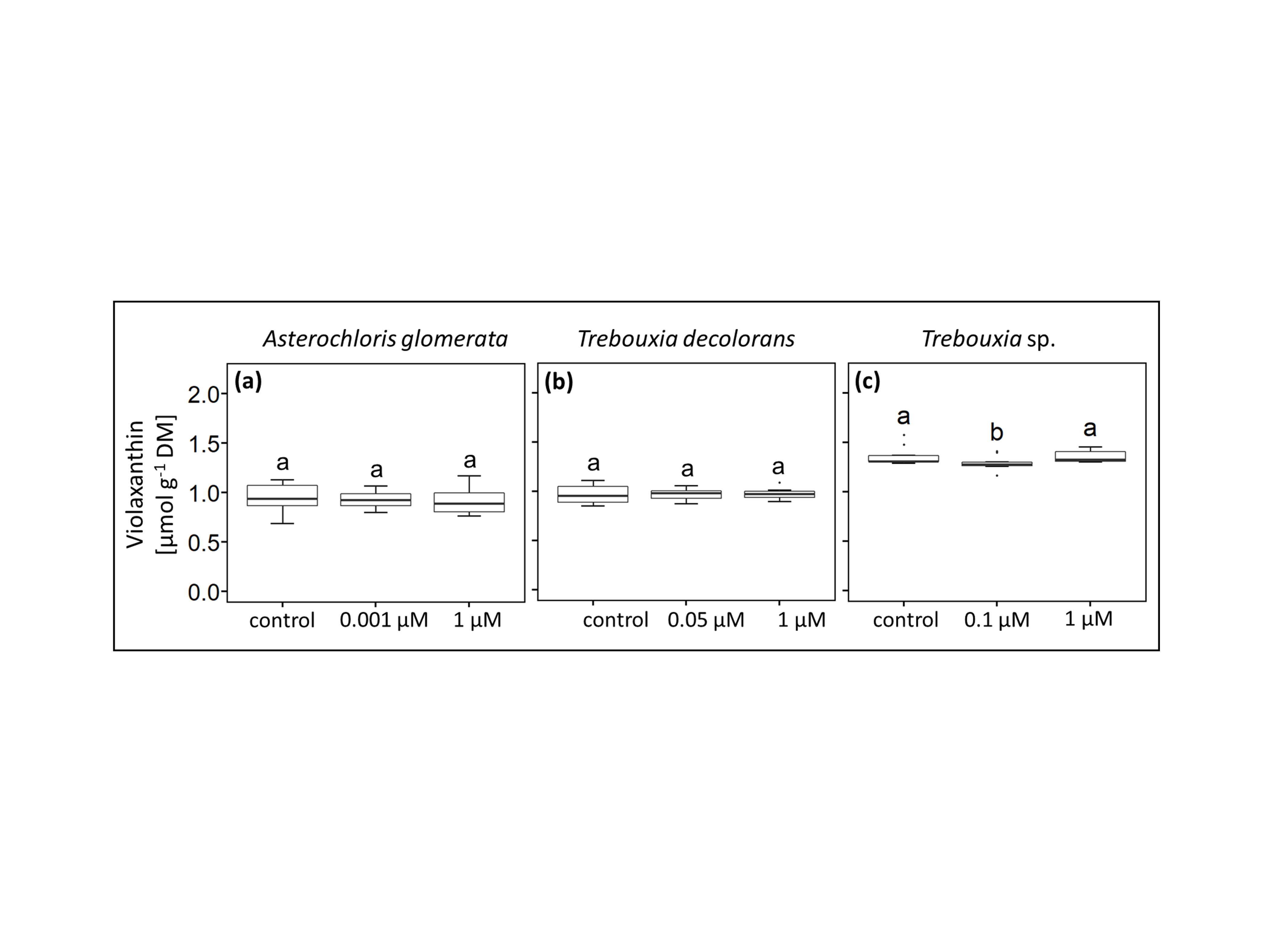

Supplement: Supplementary file 4 — High resolution image (TIF 1019 kb) [file 13199_2020_721_MOESM2_ESM.tif]
