# Supplementary material for: Phytohormone release by three isolated lichen mycobionts and the effects of indole-3-acetic acid on their compatible photobionts
Source: Symbiosis. 2020 Oct 22;82(1):95–108. doi: 10.1007/s13199-020-00721-9 (PMC7671983; doi:10.1007/s13199-020-00721-9)
Supplement: Supplementary file 5 — Contents of photosynthetic pigments of isolated photobiont cultures exposed to exogenously offered IAA. Data show mean ± SD values of chlorophyll a (Chl a) and b (Chl b), lutein (L), zeaxanthin (Z), antheraxanthin (A), violaxanthin (V), α-carotin (α-C) and β-carotin (β-C), and α-tocopherol (α-T) levels of Asterochloris glomerata, Trebouxia decolorans and Trebouxia sp. of untreated cultures (controls), and of cultures exposed to IAA exogenously applied either at physiological concentrations (0.001, 0.05 and 0.1 μM, defined by the IAA concentrations released extracellularly by their respective mycobionts) or “high” IAA concentrations (1 μM); n = 10 biological replicates. Statistically significant differences, assessed with the Kruskal-Wallis-Test (p value <0.05) are indicated by different superscript letters. (DOCX 25 kb) [file 13199_2020_721_MOESM3_ESM.docx]

| **Species** | **Chl a** | **Chl b** | **L** | **N** | **Z** | **A** | **V** | **α-C** | **β-C** | **α-T** |
| --- | --- | --- | --- | --- | --- | --- | --- | --- | --- | --- |
| Treatment | mean ± SD | | [µM g^-1^ DM] | |  |  |  |  |  |  |
| ***A. glomerata*** |  |  |  |  |  |  |  |  |  |  |
| *Control* | 9.6 ± 1.4 | 3.8 ± 0.4 | 1.7 ± 0.2 | 0.5 ± 0.1 | 0.3 ± 0.0 | 0.1 ± 0.0 | 0.9 ± 0.1 | 0.1 ± 0.0 | 0.5 ± 0.1 | 0.6 ± 0.1 |
| 0.001 µM IAA | 9.3 ± 1.0 | 3.7 ± 0.3 | 1.7 ± 0.1 | 0.5 ± 0.0 | 0.3 ± 0.0 | 0.1 ± 0.0 | 0.9 ± 0.1 | 0.1 ± 0.0 | 0.5 ± 0.1 | 0.6 ± 0.0 |
| 1 µM IAA | 9.1 ± 1.2 | 3.7 ± 0.4 | 1.6 ± 0.1 | 0.5 ± 0.1 | 0.3 ± 0.0 | 0.1 ± 0.0 | 0.9 ± 0.1 | 0.1 ± 0.0 | 0.5 ± 0.1 | 0.6 ± 0.0 |
| ***T. decolorans*** |  |  |  |  |  |  |  |  |  |  |
| Control | 9.2 ± 1.2 | 2.7 ± 0.3 | 1.6 ± 0.2 | 0.5 ± 0.0 | 0.3 ± 0.0 | 0.1 ± 0.0 | 1.0 ± 0.1 | 0.1 ± 0.0 | 0.6 ± 0.1 | 0.7 ± 0.1 |
| 0.05 µM IAA | 9.2 ± 0.7 | 2.7 ± 0.2 | 1.6 ± 0.1 | 0.5 ± 0.0 | 0.3 ± 0.0 | 0.1 ± 0.0 | 1.0 ± 0.1 | 0.1 ± 0.0 | 0.6 ± 0.1 | 0.7 ± 0.0 |
| 1 µM IAA | 9.0 ± 0.8 | 2.6 ± 0.2 | 1.6 ± 0.1 | 0.5 ± 0.0 | 0.3 ± 0.0 | 0.1 ± 0.0 | 1.0 ± 0.1 | 0.1 ± 0.0 | 0.6 ± 0.1 | 0.7 ± 0.0 |
| ***Trebouxia* sp.** |  |  |  |  |  |  |  |  |  |  |
| Control | 11.1 ± 0.8 | 3.2 ± 0.2 | 2.2 ± 0.1 | 0.6 ± 0.0 | 0.3 ± 0.0 | 0.1 ± 0.0 | 1.4 ± 0.1^a^ | < 0.1 | 0.6 ± 0.1 | 0.9 ± 0.1 |
| 0.1 µM IAA | 10.7 ± 0.6 | 3.0 ± 0.1 | 2.1 ± 0.1 | 0.6 ± 0.0 | 0.3 ± 0.0 | 0.1 ± 0.0 | 1.3 ± 0.1^b^ | < 0.1 | 0.7 ± 0.1 | 0.9 ± 0.1 |
| 1 µM IAA | 11.0 ± 0.7 | 3.2 ± 0.3 | 2.2 ± 0.1 | 0.7 ± 0.0 | 0.3 ± 0.0 | 0.1 ± 0.0 | 1.4 ± 0.1^a^ | < 0.1 | 0.6 ± 0.1 | 0.9 ± 0.1 |
